# Risk Alleles of *USF1* Gene Predict Cardiovascular Disease of Women in Two Prospective Studies

**DOI:** 10.1371/journal.pgen.0020069

**Published:** 2006-05-12

**Authors:** Kati Komulainen, Mervi Alanne, Kirsi Auro, Riika Kilpikari, Päivi Pajukanta, Janna Saarela, Pekka Ellonen, Kaisa Salminen, Sangita Kulathinal, Kari Kuulasmaa, Kaisa Silander, Veikko Salomaa, Markus Perola, Leena Peltonen

**Affiliations:** 1 Department of Molecular Medicine, National Public Health Institute, Biomedicum, Helsinki, Finland; 2 Department of Human Genetics, David Geffen School of Medicine at UCLA, University of California Los Angeles, Los Angeles, California, United States of America; 3 Department of Epidemiology and Health Promotion, National Public Health Institute, Helsinki, Finland; 4 Department of Medical Genetics, University of Helsinki, Helsinki, Finland; 5 The Broad Institute, Massachusetts Institute of Technology, Boston, Massachusetts, United States of America; University of Oxford, United Kingdom

## Abstract

Upstream transcription factor 1 (USF1) is a ubiquitously expressed transcription factor controlling several critical genes in lipid and glucose metabolism. Of some 40 genes regulated by USF1, several are involved in the molecular pathogenesis of cardiovascular disease (CVD). Although the *USF1* gene has been shown to have a critical role in the etiology of familial combined hyperlipidemia, which predisposes to early CVD, the gene's potential role as a risk factor for CVD events at the population level has not been established. Here we report the results from a prospective genetic–epidemiological study of the association between the *USF1* variants, CVD, and mortality in two large Finnish cohorts. Haplotype-tagging single nucleotide polymorphisms exposing all common allelic variants of *USF1* were genotyped in a prospective case-cohort design with two distinct cohorts followed up during 1992–2001 and 1997–2003. The total number of follow-up years was 112,435 in 14,140 individuals, of which 2,225 were selected for genotyping based on the case-cohort study strategy. After adjustment for conventional risk factors, we observed an association of *USF1* with CVD and mortality among females. In combined analysis of the two cohorts, female carriers of a *USF1* risk haplotype had a 2-fold risk of a CVD event (hazard ratio [HR] 2.02; 95% confidence interval [CI] 1.16–3.53; *p* = 0.01) and an increased risk of all-cause mortality (HR 2.52; 95% CI 1.46–4.35; *p* = 0.0009). A putative protective haplotype of *USF1* was also identified. Our study shows how a gene identified in exceptional families proves to be important also at the population level, implying that allelic variants of *USF1* significantly influence the prospective risk of CVD and even all-cause mortality in females.

## Synopsis

Better characterization of molecular events resulting in cardiovascular disease (CVD) requires elucidation of genetic background of CVD. After a CVD candidate gene is identified in family-based studies or case-control studies, population-based prospective studies are needed to demonstrate any potential impact of allelic variants on the CVD risk at the population level. This study addresses the role of different alleles of the *upstream transcription factor 1 (USF1)* gene, encoding a transcription factor and originally associated with familial combined hyperlipidemia in rare families with multiple affected individuals. The product of *USF1* regulates numerous genes of lipid and glucose metabolism, and the authors show in large population cohorts that specific alleles of *USF1* are associated with the risk of CVD and all-cause mortality among females. The study implies an interesting female-specific risk effect, and should stimulate additional studies of the sex-specific CVD risk genes in different populations.

## Introduction

The *upstream transcription factor 1 (USF1)* gene encoding USF1, a ubiquitously expressed transcription factor controlling some 40 genes [1], based on its function is an attractive candidate gene for cardiovascular disease (CVD). Initially this gene was identified as the first familial combined hyperlipidemia (FCHL) gene in rare Finnish pedigrees with multiple affected individuals having a greatly increased risk for CVD [2]. This finding was rapidly replicated in Mexican families [3], and since then *USF1* has also been associated with the metabolic syndrome and type II diabetes in study samples ascertained for these traits [4].

Thus, the combined evidence indicates a role for *USF1* in the molecular background of hyperlipidemias, yet the direct contribution of the *USF1* gene to CVD at the population level has not been addressed. To adequately evaluate the impact of a gene or a risk allele on the disease risk at the population level requires a prospective follow-up study, a golden standard in traditional epidemiology, in which risk factor(s) are measured at the beginning of the follow-up, and the diagnostic endpoints are registered as the study proceeds. We have in this study used two unique cohorts from Finland, the population in which the *USF1* gene was identified as an FCHL gene, to address the general significance of the *USF1* gene as a risk factor for CVD in population-based, prospective manner. We evaluated allelic variants of the *USF1* gene by genotyping haplotype-tagging single-nucleotide polymorphisms (htSNPs) of the *USF1* locus and assessing their association with CVD events in the two prospective cohorts. Specific alleles of the *USF1* gene proved to modify the CVD risk in women and to contribute both to CVD and mortality at the population level.

## Results

**Figure 1:**
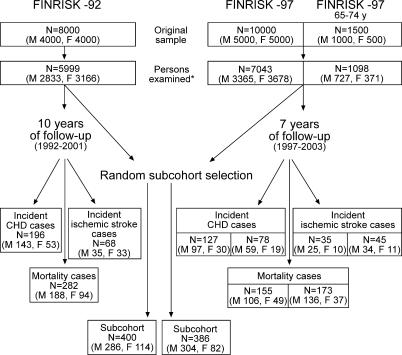
A Description of the FINRISK 1992 and 1997 Cohorts Compared to FINRISK-92, the FINRISK-97 cohort includes an additional sample of individuals aged 65–74 y. Numbers for this additional sample are described at the right-hand side for each endpoint. Persons examined refers to cohort individuals for whom information on smoking, blood pressure, cholesterol, and DNA, as well as consent for the use of DNA to study CHD and stroke, were available. Subcohorts are stratified random samples of the original cohorts including also cases. Mortality cases show total mortality, including also those who died from CHD or stroke. Thus, numbers in the boxes of subcohorts and outcome events are not mutually exclusive (see Table 1). F, females; M, males.

Figure 1 shows characteristics of the two FINRISK [5] cohorts. The number of individuals recruited in the FINRISK-97 cohort was larger than in the FINRISK-92 at the baseline of the study. However, since the follow-up time was shorter for the FINRISK-97, the number of person-years was smaller for this younger cohort (54,577 person-years in FINRISK-97 versus 57,858 person-years in FINRISK-92). We selected incident cases and random subcohorts from the FINRISK cohorts for genotyping of six htSNPs as shown in Figure 1 and Tables 1 and 2.

**Table 1:**
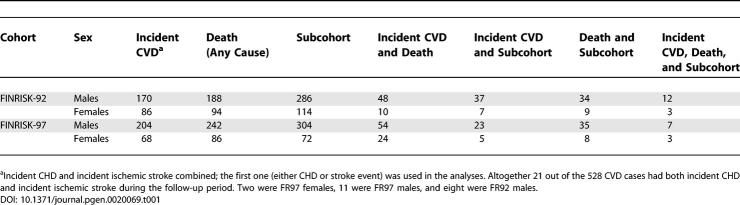
Numbers of Study Subjects in the Random Subcohort and with Different Endpoint Events in FINRISK-92 and FINRISK-97 Cohorts

**Table 2:**
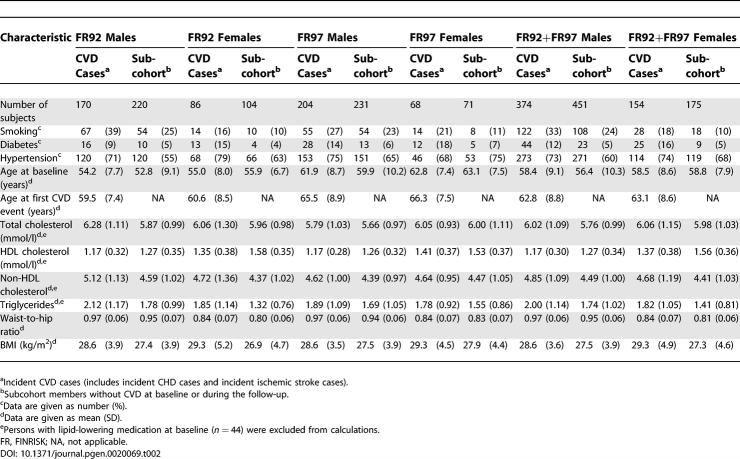
Baseline Characteristics of Cardiovascular Cases and Subcohort

**Figure 2:**
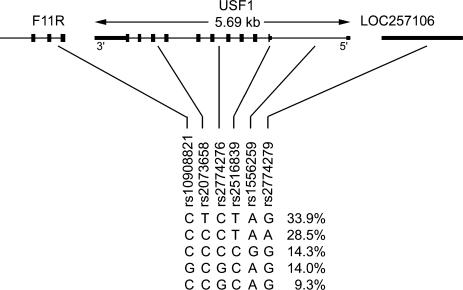
The Haplotype Structure in the *USF1* Gene Locus The six genotyped SNPs from the *USF1* gene locus resulted in five common haplotypes. The haplotype frequencies were calculated for the two FINRISK subcohorts combined.

The genotype frequencies of all six SNPs followed Hardy-Weinberg equilibrium at the 0.05 level among the subcohorts of the FINRISK study samples. The linkage disequilibrium (LD) structure of the *USF1* gene and haplotype tagging properties of the genotyped SNPs were examined combining the two subcohorts. The selected six htSNPs captured the common haplotypes of *USF1* well (all haplotypes with frequency > 4% in SeattleSNPs database (Figure 2) [6]). Due to the high LD across the *USF1* gene locus (D′ 0.995–1.000 between all SNP pairs), only five haplotypes were observed. Importantly, based on the distribution of the SNP alleles into various haplotypes, it was evident that the SNP alleles unequivocally specified haplotypes; thus, the selected SNPs truly represented htSNPs for this gene.

The minor allele frequencies of the *USF1* SNPs varied from 11% to 41% in the subcohorts (Table 3). In both FINRISK cohorts, SNP *rs2073658* showed a difference in the minor allele frequency between the female incident CVD cases and the subcohort free of CVD (*p* = 0.05 for FINRISK-92 and FINRISK-97 combined). This SNP represents the variant previously associated with FCHL in Finnish pedigrees, and in both cohorts the minor allele seemed to be more prevalent among incident female CVD cases when compared to subcohort females free of CVD. For another SNP, *rs2774279,* located 6.8 kb 5′ from *rs2073658,* the minor allele frequency was higher among the subcohort females free of CVD than among incident female CVD cases (*p* = 0.03 for FINRISK-92 and FINRISK-97 combined), especially in the FINRISK-92 (*p* = 0.008) study sample with a longer follow-up. The minor alleles of these two SNPs both tag one haplotype of *USF1*. The minor allele of *rs2073658* tags the most common *USF1* haplotype, and the minor allele of *rs2774279* tags the second most common haplotype of *USF1* (Figure 2).

**Table 3:**
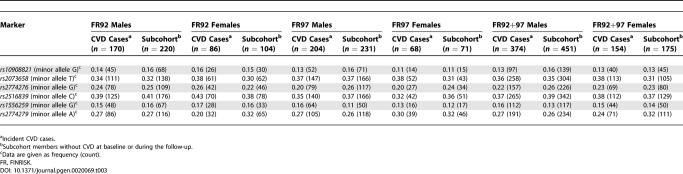
Minor Allele Frequencies of *USF1* htSNPs for CVD Cases and the Subcohort

Cox proportional hazards regression analysis measuring time-to-event was used to estimate the risk of an incident CVD event or mortality during the follow-up period in relation to the *USF1* SNPs. Allele-specific hazard ratios for the six SNPs tested are shown in Table 4 for analyses testing the two FINRISK cohorts separately and for analyses pooling the cohorts. Analysis of the previously analyzed SNP *rs2073658* [1–4,7,8] revealed an increased risk of incident CVD (HR 2.02; 95% confidence interval [CI] 1.16–3.53; *p* = 0.01) and all-cause mortality (HR 2.52; 95% CI 1.46–4.35; *p* = 0.0009) for carriers of T-allele among females when compared to noncarriers of the allele. The increased risk was evident especially among the FINRISK-97 females; however, the hazard ratio was increased also among the FINRISK-92 females, although it did not reach statistical significance. The second SNP showing a difference in the minor allele frequency between incident cases and subcohort free of CVD, *rs2774279,* was also associated with increased risk of incident CVD and mortality in time-to-event analysis. Risk of mortality was increased among the female carriers of the G-allele of *rs2774279* (HR 4.43; 95% CI 1.58–12.40; *p* = 0.005). Again, the increased risk was observed especially among the FINRISK-97 females. Also, the risk of CVD was higher among female carriers of the *rs2774279* G-allele (HR 4.01; 95% CI 1.30–12.39; *p* = 0.02), and the effect of the SNP was observed in both FINRISK cohorts. Among FINRISK-97 females, the effect of SNP *rs2774279* was visible when comparing carriers of the G-allele with others (HR 15.38; 95% CI 2.49–94.87; *p* = 0.003). Interestingly, among the FINRISK-92 females, the effect of SNP *rs2774279* was observed when comparing carriers of the A-allele with others (HR 0.33; 95% CI 0.13–0.85; *p* = 0.02).

**Table 4:**
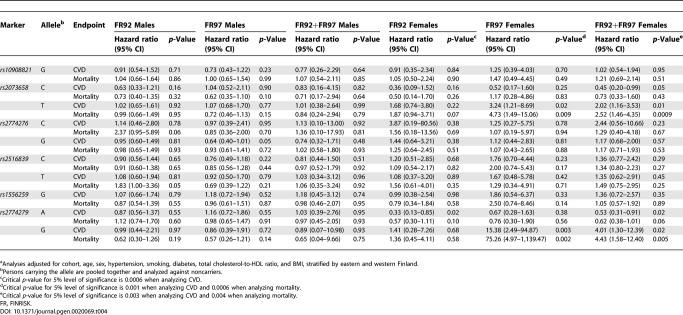
Risk of a Cardiovascular Event and All-Cause Mortality for the *USF1* SNPs^a^

No significant associations with the risk of an incident CVD event or mortality were observed among males in combined analysis of the two cohorts. Among FINRISK-92 males, a suggestive association was obtained for risk of mortality and the T-allele of SNP *rs2516839* (HR 1.83; 95% CI 1.00–3.36; *p* = 0.05), and including FINRISK-92 females to the analysis further strengthened the results (HR 1.86; 95% CI 1.13–3.05; *p* = 0.01). Otherwise, the only association seen in the pooled analyses of males and females was a decreased risk of CVD (HR 0.53; 95% CI 0.30–0.95; *p* = 0.03) observed for carriers of the *rs2073658* C-allele when compared to noncarriers of the allele among the FINRISK-92 cohort.

We used permutation analysis to determine a critical *p*-value for 5% level of significance corrected for multiple testing for our results; we permuted the genotype while retaining the phenotype data and repeated the same analyses that were performed with the actual dataset and repeated this procedure 1,000 times. In the time-to-event analyses where FINRISK-92 males and females were pooled together, permutation analysis suggested a critical *p*-value of 0.005 for a 5% level of significance for both CVD risk and mortality risk. All other critical *p*-values obtained from permutation analysis can be seen in Table 4.

In our age-stratified study samples, the average age of the onset of CVD was about the same for males and females (Table 2). Since females generally have their first CVD event at an older age than men, we tested whether the observed sex difference was primarily due to a possible difference in relative age at event for males and females in our study sample. The time-to-event analysis combined for the two cohorts was repeated for males who had their first CVD event early (before age 55; *n* = 70), but no associations with CVD or mortality were observed (unpublished data).

The time-to-event results of individual htSNPs suggested that the *rs2073658* T-allele and *rs2774279* G-allele increase the risk of CVD and mortality among females. From the *USF1* haplotypes it was obvious that the *rs2073658* T-allele was present only in haplotype CTCTAG (population frequency 34%) (Figure 2), thus specifying it as a risk-increasing haplotype. The *rs2774279* G-allele was present in all other haplotypes but the CCCTAA haplotype (population frequency 29%), thus implying a protective role for this haplotype. We wanted to investigate more closely how the carriership of these haplotypes relates to the risk of CVD and mortality. Time-to-event analysis was performed to estimate the risk of CVD for females carrying the risk haplotype CTCTAG without the protective haplotype CCCTAA. The females carrying the risk haplotype CTCTAG, but without the protective haplotype CCCTAA, had the highest CVD risk when compared to any other group of haplotype carriers**,** this risk being 2.8-fold (HR 2.77; 95% CI 1.50–5.13; *p* = 0.001). This result remained significant after correcting for multiple testing: In permutation analysis, the critical *p*-value for 5% significance level was 0.003.

Finally, we tested whether the sequence variants of *USF1* have an effect on lipids, body mass index (BMI), or waist-to-hip circumference ratio, the traits characteristic of FCHL and other conditions with earlier-reported associations with *USF1* and known contributors to the CVD risk. The obtained results support previous findings of the best association observed for lipid values in males, and are summarized in Tables 5 and S1.

**Table 5:**
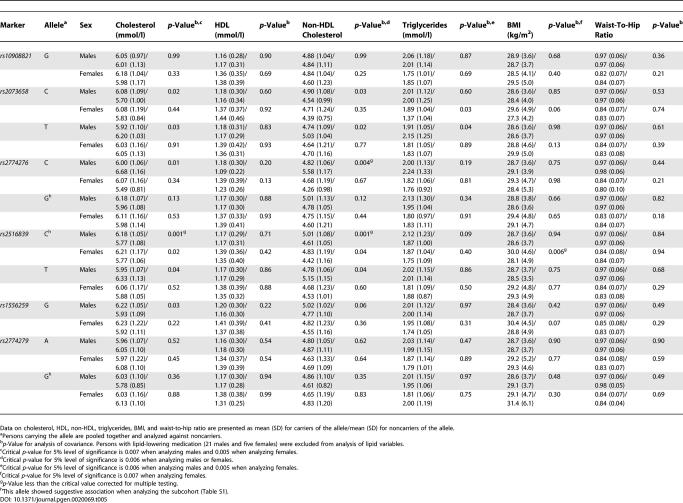
Association between *USF1* SNP Alleles and Lipids, BMI, and Waist-To-Hip Ratio in FINRISK CVD Case Males and Females

When males and females were analyzed together, significant associations among incident CVD cases were seen with higher levels of cholesterol and non-high-density lipoprotein (HDL) cholesterol with the C-allele of *rs2073658* (*p* = 0.02 and *p* = 0.02 for cholesterol and non-HDL cholesterol, respectively), C-allele of *rs2516839* (*p* < 0.0001 and *p* = 0.0001), and G-allele of *rs1556259* (*p* = 0.01 and *p* = 0.03). Among the subcohort free of CVD, the G-allele of *rs2774276* was associated with higher waist-to-hip ratios (*p* = 0.009). For all these pooled analyses the critical *p*-value for 5% significance level was 0.006.

## Discussion

We previously established the association of the *USF1* gene with FCHL in a set of rare Finnish families with multiple dyslipidemic individuals [2], known to be predisposed to a significantly increased risk for CVD. Here we show that allelic variants of the *USF1* gene have an impact on CVD risk also at the population level, a finding rarely established for any complex disease gene so far.

The biological importance of the *USF1* gene has been implied in several studies reporting an association between *USF1* and disorders of lipid and glucose metabolism, mostly in study samples ascertained for these traits. Following the first association report for *USF1* with FCHL in Finnish FCHL families [2], the association was replicated in Mexican FCHL families [3]. Further, in Utah pedigrees ascertained for early death due to coronary heart disease (CHD), early stroke, or early-onset of hypertension, *USF1* was found to be associated with FCHL-related lipid traits, especially in males [8]. *USF1* has also been implicated in the etiology of metabolic syndrome and type II diabetes [4], common conditions predisposing to premature CVD. Additional studies show variants of *USF1* as being associated with features of glucose and lipid homeostasis in healthy young men [9] and increased adipocyte lipolysis in healthy obese women [7]. Although multiple studies have provided evidence of the role of *USF1* in CVD-associated dyslipidemias, the direct contribution of the *USF1* gene to CVD and mortality at the population level has not been assessed.

Here we prospectively followed up two distinct cohorts representative of the general Finnish population of the study areas. Using Cox proportional hazards regression analysis measuring time-to-event, we found a female-specific association of two *USF1* htSNPs, defining a “risk allele” of *USF1,* with both CVD and all-cause mortality. We also tentatively identified a “protective” allele for this gene.

The *USF1* risk allele identified here for the cohort females differs from the risk allele segregating in FCHL families. In 60 Finnish FCHL families the risk was associated with the common allele of *rs2073658* [2], whereas in the present study the SNP haplotype CTCTAG, carrying the minor allele of *rs2073658,* was associated with a risk effect. However, our original FCHL study did not address the female risk allele in any detail, since the most significant association was observed in males with high triglycerides. We performed a simple analysis of the original FCHL data, choosing families (32 out of 60) in which at least 50% of those affected were female. Among these affected FCHL females the frequency of the minor allele of *rs2073658* (haplotype CTCTAG seen in FINRISK females) was higher than among the unaffected females of those families (45% versus 38%, respectively), thus supporting our findings. In addition, the frequency of the “protective” FINRISK haplotype CCCTAA was 23% in affected females of those families and 30% in unaffected females. Thus the obtained results of the “risk allele” of *USF1* in these two studies are comparable, although we recognize that the ascertainment strategy for the FCHL families results in a biased selection of population alleles and complicates the estimation of any sex-specific effect in these rare families. Many previous studies have found the influence of *USF1* to be more prominent among males [2,8], or have studied only males [9], whereas here we saw the most significant evidence of association among females. The lack of association among males at the population level in our cohorts could be explained by environmental covariates. CVD events are more common in males, most probably due to clustering of numerous lifestyle risk factors, including smoking, obesity, and high blood pressure, this increasing the environmental “noise” and complicating genetic analyses in males compared to females.

The association of *USF1* with CVD in females of two distinct population cohorts supports the real involvement of *USF1* in CVD risk. Moreover, the trends of the hazard ratios for the risk-associated alleles of *USF1* were the same in both FINRISK cohorts, and the same risk alleles of *USF1* were associated both with CVD events and all-cause mortality among females, further supporting their significance. Finally, correcting for multiple testing with permutations providing a more stringent significance threshold for the original *p*-values still retained a significant association between *USF1* variation and the risk of CVD and mortality among females.

In the association analyses of SNPs with lipid parameters, we observed *USF1* htSNPs to strongly associate with plasma cholesterol levels and non-HDL cholesterol especially among the male CVD cases. These findings confirm prior reports that *USF1* variants contribute to differences in lipid profiles [2–4,7–9]. However, no associations with traditional risk factors were obtained that would explain the increased risk of CVD and mortality among female carriers of *USF1* risk alleles. The question remains whether the impact of *USF1* on lipid parameters among females is as powerful as among males, or if the gene variants have alternative ways of increasing the CVD risk—for example, through inflammation-related pathways.

As noted previously, the strong LD of the *USF1* locus extends the critical FCHL region to 46 kb in Finnish FCHL families [2]. This DNA region may harbor additional variants that represent true functional variants that affect CVD risk. Sequencing of the complete region in sufficiently large study samples would shed light on the risk-increasing variants of *USF1,* and would enable the detailed functional studies needed to establish their role. So far, functional data exist only for the most significantly associated SNP of the original FCHL study, *rs2073658* [1].

In conclusion, here we have shown that the risk effect of a gene identified in a rare set of FCHL families who are at exceptionally high risk for cardiovascular events can be confirmed in a population-based prospective follow-up study. We observed one “risk” and one “protective” allelic haplotype of *USF1* that significantly contribute to the risk for CVD and all-cause mortality among females. To evaluate the significance of these results obtained in the Finnish cohorts for CVD risk more globally, other populations need to be analyzed. Still, this study demonstrates, to our knowledge for the first time, that *USF1* gene variation may have a long-term predictive effect on CVD risk in females, and thus adds another piece of proof to the accumulating evidence that the gene plays an important role in the progression of CVD.

## Materials and Methods

### Study populations.

Our population cohorts were collected via the FINRISK [5] studies. FINRISK surveys are carried out every 5 y and are designed to assess the prevalence and risk factors of cardiovascular diseases in Finland. The FINRISK-92 study contacted a random sample of 8,000 persons aged 25–64 y from four geographical areas of Finland (Helsinki region, southwestern Finland, North Karelia, and Kuopio region) as part of the WHO MONICA Project (Multinational Monitoring of Trends and Determinants in Cardiovascular Disease, an international study conducted under the auspices of the World Health Organization) [10]. The persons were selected through random sampling of the population, stratified by sex, area, and 10-y age group (25–34, 35–44, 45–54, and 55–64 y). The protocol was as established by the WHO MONICA [10] Project.

The FINRISK-97 study contacted 10,000 persons from the same regions, selected using the same sampling procedure and using the same protocols as the FINRISK-92 study, but including also the province of Oulu. In addition, 250 females and 500 males aged 65–74 y were included from two of the areas. At the beginning of the follow-up period, the overall response rates covering participants with all required information for the genetic study were 71% for males and 79% for females in the FINRISK-92 study, and 68% for males and 74% for females in the FINRISK-97 study, resulting in a total of 14,140 participants out of the 19,500 in the original sample (Figure 1). No overlap exists between the FINRISK-92 and the FINRISK-97 cohorts.

The FINRISK-92 and FINRISK-97 studies were approved by the Ethical Committee of the National Public Health Institute, the participants gave an informed consent, and the principles of the Helsinki Declaration were followed.

Both FINRISK-92 and −97 cohorts are participating in the ongoing MORGAM Project (http://www.ktl.fi/morgam) [11], a multinational collaborative study.

The follow-up period for participants of FINRISK-92 lasted from spring 1992 until the end of December 2001, a total of 10 y. In FINRISK-97 the participants were followed up for 7 y, from 1997 until the end of December 2003. More detailed information about the FINRISK cohorts and the follow-up procedures can be found in the cohort descriptions of the MORGAM Project (http://www.ktl.fi/publications/morgam/cohorts).

### Baseline examination and follow-up of FINRISK studies.

At the baseline, all participants were asked to fill in a self-administered questionnaire and were physically examined. The physical examination included blood pressure, waist, hip, weight, and height measurements. The self-administered questionnaire asked respondents for information on the most established environmental cardiovascular risk factors, e.g., demographic variables, history of CVD, history of diabetes, medication, smoking, and additional lifestyle and dietary habits. Blood was collected in a “semifasting” state, i.e., the participants were instructed to fast for 4 h and to avoid fatty meals earlier during the day. Serum total cholesterol, HDL cholesterol, and triglyceride levels were measured. Whole blood was stored at −20 °C for DNA extraction.

### Case-cohort design.

We used a case-cohort design [12], in which DNA was genotyped only for stratified random samples—subcohorts—of the FINRISK-92 and −97 cohorts, and for participants experiencing death, coronary event, or stroke during the follow-up periods (Figure 1). In addition, DNA of participants experiencing venous thromboembolic event or reporting history of CVD at the baseline examination was genotyped (http://www.ktl.fi/publications/morgam/cohorts); however, venous thromboembolism and baseline CVD were not analyzed as endpoints in our genetic study. Further, participants with prevalent CVD were excluded from our genetic analyses, except when analyzing all-cause mortality, for which all subcohort members and all deaths were included regardless of their CVD history. Information about coronary and stroke events during the follow-up periods was obtained from several sources: specific myocardial infarction and stroke registers (FINAMI [13] and FINSTROKE [14]) complemented with record linkage of the study data with the National Causes of Death Register and the National Hospital Discharge Register. These country-wide, computerized registers cover every death of Finnish citizens and every hospitalization in Finland, and thus the coverage of follow-up for symptomatic CVD events was almost 100%. In the National Causes of Death Register International Classification of Diseases (ICD)-9 codes 410–414 and 798, and ICD-10 codes I21–I25, I46, R96, R98 and R99 were taken as coronary deaths. ICD-9 codes 433 (excluding 4330X, 4331X, 4339X of the Finnish modification of ICD-9; see http://www.ktl.fi/publications/morgam/cohorts) and 434 (excluding 4349X), and ICD-10 code I63, were taken as ischemic stroke deaths. In the National Hospital Discharge Register ICD-9 codes 410–411, and ICD-10 codes I21–I22 and I20.0, were taken as nonfatal coronary events; and ICD-9 codes 433 (excluding 4330X, 4331X, 4339X) and 434 (excluding 4349X), and ICD-10 code I63, were taken as nonfatal ischemic stroke events. ICD-9 was used in Finland until 31 December 1995, and ICD-10 after that. The combination of FINAMI register and the National Hospital Discharge Register was also used to identify revascularization procedures (CABG and PTCA) during the follow-up period. The validity of cardiovascular diagnoses in the Finnish Causes of Death Register and the Hospital Discharge Register has been documented in several publications [15–17].

Unique Finnish social security numbers of the participants were compared between the two cohorts, and participants already enrolled in the FINRISK-92 cohort were excluded from the FINRISK-97 cohort.

In the present study, incident CVD and all-cause mortality were analyzed as endpoints. Incident CVD cases included the persons without prevalent CVD at baseline who suffered a fatal or nonfatal ischemic stroke event, or fatal or nonfatal coronary event during the follow-up period (Figure 1 and Table 1). The number of main study endpoints was used as reference to determine the size of the random subcohorts to be selected from the original population cohorts and age was controlled through sampling weights (http://www.ktl.fi/publications/morgam/manual/contents.htm) (Figure 1). The selection of the subcohort members was independent of the selection of cases, thus of the 374 male and 154 female incident CVD cases, 72 were also part of the subcohorts. Altogether 610 persons (180 females and 430 males) died during the follow-ups of the two cohorts. Table 2 shows the baseline characteristics of the incident CVD cases and subcohort members free of CVD.

### DNA extraction.

DNA was extracted from EDTA-treated whole-blood samples using a standard phenol-chloroform method modified from Vandenplas et al. [18]. DNA of 23 FINRISK-92 samples was extracted by a salt-precipitation method.

### Markers.

To capture the allelic diversity of the 5.69 kb *USF1* gene in the FINRISK-92 and −97 cohorts, we genotyped six htSNPs (Figure 2). *Rs2073658* in intron 7 of the *USF1* gene was the most significantly associated SNP in our previous FCHL study [2]. Additional htSNPs were selected using the SeattleSNPs Variation Discovery Resource [6]. All common (average minor allele frequency > 4%) LD select bins for European descent were covered with our SNP selection.

*Rs2516839* and *rs1556259* were included in our family-based study [2], but showed no association with FCHL. *Rs10908821* is located in an intron of the F11 receptor gene *(F11R),* 2.23 kb downstream from *rs2073658*. *Rs2774276* resides in intron 5 of *USF1,* 0.95 kb upstream of *rs2073658*. SNP *rs2774279* results in a synonymous amino acid substitution (874 R/R) in an exon of a predicted gene LOC257106, and is located 2.90 kb upstream from *rs1556259*.

### Genotyping.

Most of the genotyping was performed using the MassARRAY System (Sequenom, San Diego, California, United States), with a protocol described elsewhere [19]. SNPs *rs2073658, rs2516839,* and *rs2774279* were genotyped in the FINRISK-92 study sample using allele-specific primer extension on microarrays with a protocol described elsewhere [19,20]. For MassARRAY System genotyping, 81 low-yield DNA samples of the FINRISK-92 study sample and 19 low-yield DNA samples of the FINRISK-97 study sample were first amplified in replicates using GenomePhi DNA amplification kit (GE Healthcare UK, Buckinghamshire, England) as specified in the manufacturer's instructions and genotyped as described [19].

The staff in the genotyping laboratory were blinded to sex and disease status of participants. To control for sample mix-ups, all samples were genotyped for sex, and the data were compared to the sex of participants as recorded in the database. Plate-specific duplicates and water samples were used to control for plate-handling errors. Of the samples genotyped, 5% were blinded duplicates. The *USF1* marker genotypes of the blinded duplicate pairs were compared after genotyping and were all found to be consistent. The success rate of genotyping was 98% for SNP *rs2774276* and 97% for all other SNPs.

### Haplotype estimations.

LD and haplotype block analysis and haplotype population frequency estimations were performed with Haploview version 3.2 [21] using the two FINRISK subcohorts combined. Subcohort members with over 50% missing genotypes were excluded from the analysis, thus 782 individuals were used in the haplotype estimations.

### Data analyses.

Allele and genotype frequencies were determined from the data, and deviation from the Hardy-Weinberg equilibrium was tested using Pearson's chi-square. Pearson's chi-square statistic was used to compare allele frequencies between incident CVD cases and subcohorts free of CVD.

A weighted Cox proportional hazards model, modified to account for the case-cohort sampling design, was used for risk analyses with the variance correction based on the published literature using SAS PHREG procedure [12,22,23]. Age represented the time measure in the models. Covariates known to associate with CVD (age, sex, hypertension, smoking, diabetes, total cholesterol to HDL ratio, and BMI) were included in multivariate models as potential confounders. When analyzing CVD, individuals with prevalent CVD at the baseline were excluded from analyses. Analyses were stratified for eastern and western Finland, and the cohort was used as a covariate. Carriership of the minor and major allele was analyzed for each SNP, except for the two SNPs with lowest minor allele frequencies *(rs10908821* and *rs1556259),* for which only the carriership of the minor allele was used in the analysis due to the low number of individuals homozygous for the minor allele.

To determine the relation between the *USF1* htSNPs and several baseline variables, we computed age-, sex-, geographical area-, and cohort-adjusted mean values for SNP allele groups and tested the difference with analyses of covariance implemented in the SAS “PROC GLM” procedure. Before analyses, the variables HDL and triglyceride were log-transformed to correct them to be normally distributed. Mean values of the variables are displayed as crude data on Tables 5 and S1. Individuals deviating ± 4 standard deviations (SD) from the mean were excluded as outliers from the analyses. Further, the analyses were conducted using subcohort members free of CVD at baseline and incident CVD cases.

To evaluate the significance of our findings, we permuted the genotype while retaining the phenotype data within sex/cohort groups, and repeated the same analyses that were performed with the actual dataset, recording the minimum *p*-value observed. We repeated this procedure 1,000 times and took the fifth percentile of these minimum *p*-values as the new multiple-testing corrected threshold for the *p*-values obtained with the original data.

Statistical analyses were carried out using the SAS statistical software (versions 8.2. and 9.1 for Windows) (SAS OnlineDoc, Version 8, 1999; SAS Institute, Cary, North Carolina, United States).

## Supporting Information

Table S1 Association between *USF1* SNP Alleles and Lipids, BMI, and Waist-To-Hip Ratio in FINRISK Subcohort Males and Females (27 KB XLS)

### Accession Numbers

The Entrez Gene (http://www.ncbi.nlm.nih.gov/entrez/query.fcgi?db=gene) accession numbers for *USF1* and *F11R* are 7391 and 50848, respectively. The online Mendelian Inheritance in Man (OMIM) http://www.ncbi.nlm.nih.gov/entrez/query.fcgi?db=omim) accession numbers for FCHL, metabolic syndrome, and type II diabetes are 144250, 605552, and 125853, respectively.

## Abbreviations

BMI: body mass index
CHD: coronary heart disease
CI: confidence interval
CVD: cardiovascular disease
FCHL: familial combined hyperlipidemia
HDL: high-density lipoprotein
HR: hazard ratio
htSNP: haplotype-tagging single nucleotide polymorphism
ICD: International Classification of Diseases
LD: linkage disequilibrium
SD: standard deviation
SNP: single nucleotide polymorphism
USF1: upstream transcription factor 1
